# Exosomal cargos-mediated metabolic reprogramming in tumor microenvironment

**DOI:** 10.1186/s13046-023-02634-z

**Published:** 2023-03-10

**Authors:** Shiming Tan, Yiqing Yang, Wenjuan Yang, Yaqian Han, Lisheng Huang, Ruiqian Yang, Zifan Hu, Yi Tao, Lin Liu, Yun Li, Linda Oyang, Jinguan Lin, Qiu Peng, Xianjie Jiang, Xuemeng Xu, Longzheng Xia, Mingjing Peng, Nayiyuan Wu, Yanyan Tang, Deliang Cao, Qianjin Liao, Yujuan Zhou

**Affiliations:** 1 Hunan Key Laboratory of Cancer Metabolism, Hunan Cancer Hospital and the Affiliated Cancer Hospital of Xiangya School of Medicine, Central South University, Changsha, 410013 Hunan China; 2 University of South China, Hengyang, 421001 Hunan China; 3 Hunan Key Laboratory of Translational Radiation Oncology, 283 Tongzipo Road, Changsha, 410013 Hunan China

**Keywords:** Exosomal cargo, TME, Metabolism, CAFs, Angiogenesis, T lymphocytes, TAMs, Adipocytes, Stellate cells, ECM

## Abstract

Metabolic reprogramming is one of the hallmarks of cancer. As nutrients are scarce in the tumor microenvironment (TME), tumor cells adopt multiple metabolic adaptations to meet their growth requirements. Metabolic reprogramming is not only present in tumor cells, but exosomal cargos mediates intercellular communication between tumor cells and non-tumor cells in the TME, inducing metabolic remodeling to create an outpost of microvascular enrichment and immune escape. Here, we highlight the composition and characteristics of TME, meanwhile summarize the components of exosomal cargos and their corresponding sorting mode. Functionally, these exosomal cargos-mediated metabolic reprogramming improves the "soil" for tumor growth and metastasis. Moreover, we discuss the abnormal tumor metabolism targeted by exosomal cargos and its potential antitumor therapy. In conclusion, this review updates the current role of exosomal cargos in TME metabolic reprogramming and enriches the future application scenarios of exosomes.

## Background

Extracellular vesicles (EVs) are nanoscale cellular secretions that act as key mediators in many pathological/physiological processes [1, 2]. According to MISEV2018, EVs cover a variety of subtypes such as exosomes, microvesicles (MV, also known as microparticles and ectosomes) and apoptotic bodies [3–6]. In addition, some emerging subtypes of EVs have gained increasing attention, such as two non-membrane nanoparticles exomere and supermere [7, 8]. A bilayer membrane vesicle, called migrasome, generated at the end or crossover site of contractile filaments produced by the cell tail during directed cell migration, has gradually attracted interest since its discovery in 2014 [9, 10]. On this basis, the investigators further identified mitosomes, defined as migrasomes that contain mitochondria [11]. The field of EVs is currently in a developmental stage, in this review we will elaborate on the role of exosomes, a classical subtype of EVs, in the regulation of metabolic reprogramming in the tumor microenvironment around exosomes. (Table. 1).

**Table 1:** Major subtypes of EVs and their physiological characteristics

EVs subtype	Characteristics				Reference
	Size (nm in diameter)	Membrane	Origin	Markers	
Exosomes	40–160	Double	ILVs	CD9, CD63, CD81, HSP70, TSG101, flotillin-1, ALIX	[12]
Microvesicles	100–1000	Double	Plasma membrane	Selectins, intergrins, CD40, ARF6	[6]
Apoptotic bodies	50–5000	Double	Plasma membrane (during apoptosis)	C1q, ICAM-3, clathrin, Calreticulin, CD44v6	[5]
Exomeres	< 50	Non	Separation from exosomes	Hsp90-b,	[7]
Supermeres	Around half the volume of exomere	Non	Separation from exomeres	TGFBi	[8]
Migrasomes	500–3000	Double	Retraction fibres (during migration)	NDST1, PIGK, CPQ, EOGT	[10]
Mitosomes		Double	Separation from migrasomes		[11]

Exosomes are nanoscale bilayered vesicles that are actively released by cells into the extracellular fluid and carry a variety of genetic materials [13]. They are taken up by cells through autocrine or paracrine pathways, and can also be taken up by distant tissues or organs via the circulatory system, participating in a variety of physiological and pathological processes [14, 15]. Initially, exosomes were thought to be "redundant" substances released by cells, but with the progress in the field of "regulation of cellular vesicular transport", exosomes have gradually become a hot topic in basic and translational medicine [16].

Exosomes carry host cell-derived proteins, lipids, non-coding RNA (ncRNA) and metabolites. These bioactive substances can be involved in regulating intercellular communication between tumor cells and TME and mediating TME heterogeneity [17, 18]. Exosomal cargos participate in processes, such as deregulating cellular energetic, avoiding immune destruction and sustained angiogenesis [17, 19–21]. The presence of differentially expressed exosomal proteins, ncRNAs and metabolites profiles in many solid tumors suggests a potential clinical diagnostic and therapeutic value of exosomal cargos.

TME is a "sanctuary" for tumor cells. Previously, the primary goal of human tumor treatment was the direct elimination of tumor cells. With the introduction of the TME concept, tumor is no longer composed of aggregates of tumor cells, but of surrounding cells and non-cellular components [22]. TME not only provides the space and conditions for tumor cells to survive, but on the contrary, tumor cells can also modify TME by secreting exosomal cargos. By remodeling the metabolic pathways of non-tumor cells in TME, exosomal cargos can promote TME heterogeneity and provide a precursor to tumor recurrence or distant metastasis [23].

## Main text

### Main components of the tumor microenvironment

**Fig. 1:**
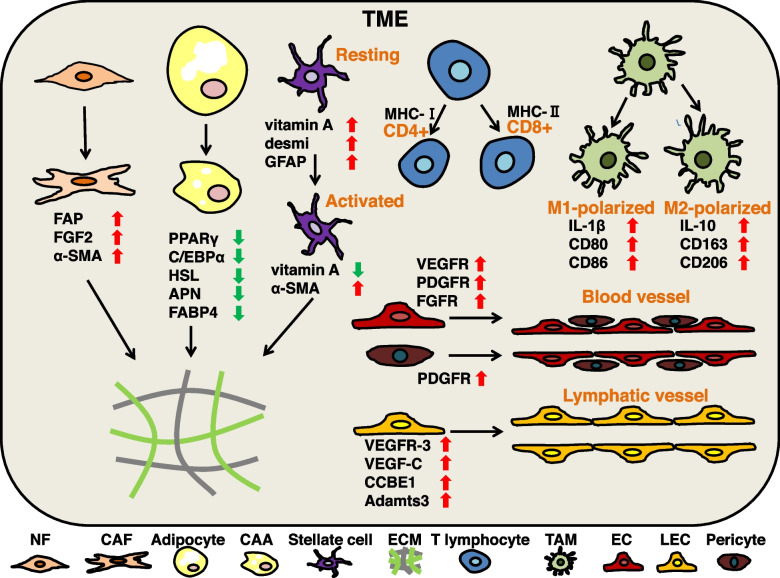
Mainly components of the TME and their biological characteristics. In the TME, NFs can differentiate into CAFs with highly expression of FAP, FGF2 and α-SMA. Adipocytes can differentiate into CAAs with low expression of PPARγ, C/EBPα, HSL, APN and FABP4. Resting stellate cells are rich in vitamin A, with highly expression of desmi and GFAP, whereas activated stellate cells lack vitamin A, with highly expression of α-SMA. CAFs, CAAs and stellate cells produce ECM by secreting components such as collagen. According to the different MHC molecules, T lymphocytes can activate into two main subtypes, CD4 + T or CD8 + T cells. M1-polarized TAMs can delay tumor progression with highly expression of IL-1β, CD80 and CD86. M2-polarized TAMs benefit to tumor progression with highly expression of IL-10, CD163 and CD206. Tumor microvascular tissue consists of ECs and pericytes. VEGFR and FGFR promote the maturation and migration of ECs. PDGFR can maintain pericyte stability the stability of pericytes. Up-regulated CCBE1, Adamts3, VEGFR-3 and its ligand VEGF-C favor lymphangiogenesis in TME

TME consist of tumor cells, resident and recruited host cells (mainly CAFs (cancer-associated fibroblasts) and immune cells), secreted products of the above cells (such as cytokines, chemokines), vasculature and ECM (extracellular matrix) [24, 25]. Specific metabolites (lactic acid, polyamines, and nitric oxide) are also present in TME [26]. These complex components contribute to the malicious behavior of TME in local resistance, immune escape and distant metastasis. (Fig. 1).

#### CAFs

CAFs are one of the main cell types involved in ECM remodeling in TME, with highly heterogeneity [27]. It is notable that CAFs are activated by normal fibroblasts (NFs) residing in the local microenvironment. Up-regulated FAP, FGF2 and α-SMA can promote the activation and heterogeneity of CAFs [28]. Activated CAFs contribute to the remodeling of ECM, which is conducive to tumor cell migration, invasion and treatment resistance [29]. In addition, the growth factor and chemokine secreted by CAFs are conducive to angiogenesis and the recruitment of immune cells [25, 30].

A previous report has proved the heterogeneity of CAFs. Researchers have characterized four CAF subtypes with different characteristics and activation levels in breast cancer. Costa et al. distinguished CAF into different subtypes (CAF-S1, 2, 3 and 4) based on the expression levels of CD29, FAP, α-SMA, PDGFRb, FSP1 and CAV1, among which CAF-S1 and CAF-S4 fibroblasts expressed a-SMA and were regarded as myofibroblasts [31]. In addition, CAF-S1 subset plays a crucial part in immunosuppression by increasing the survival rate of CD4 + CD25 + T lymphocytes and promoting their differentiation into CD25 + FOXP3 + cells, while CAF-S4 subset has no such activity [32]. Four CAF subtypes have also been identified in high-grade serous ovarian cancer (HGSOC). The expression of CXCL12β in CAF-S1 subset inhibits immune activity and is a reliable prognostic factor in HGSOC [33]. Further research found that CAF-S1 and CAF-S4 subsets accumulate in LN and are related to cancer cell invasion in the metastatic lymph nodes of breast cancer [34]. CAF-S4 subset promotes cancer cell invasion through NOTCH signal transduction, and patients with CAF-S4 subset are prone to distant metastasis [34]. These reports indicate that the heterogeneity of CAFs is closely related to the malignant phenotypes of TME.

#### TAMs

Tumor-associated macrophages (TAMs) are the major infiltrating immune cells in TME, with high heterogeneity and plasticity [35]. In the process of tumor formation, the proliferation of macrophages residing in the macrophage pool and the recruitment of monocyte-derived macrophages (MDM) to TME together represent TAM [36]. Generally, under the stimulation of different physiological and pathological factors, macrophages can differentiate into two phenotypes: classically activated (M1) and alternatively activated (M2) [37]. Among them, M1 participates in the pro-inflammatory response and also plays an anti-tumor effect, while M2 participates in the anti-inflammatory response and promotes tumor development through tumor immunosuppressive effects [38]. However, given the heterogeneity of TAMs, the M1/M2 dichotomy is no longer sufficient to explain the polarization of macrophages in TME. With the deepening of research, people are now more focused on the dynamics of the TAM polarized ecosystem [39]. Researchers have used NanoString gene expression profiles to show that TAMs in the peripheral blood of renal cell carcinoma (RCC) patients with does not completely conform to the traditional M1-M2 TAMs paradigm [40]. Some researchers have used a boolean model of macrophage polarization to simulate the activation of the corresponding phenotypes of M1, M2 and Nurse Like Cells to promote tumor activity [41]. More importantly, the heterogeneity of TAMs is not only reflected in the physical development of tumors, but there is significant heterogeneity in TAMs in autochthonous murine treated with two different treatment methods (molecular-targeted inhibitor and radiation) [42]. Therefore, the dynamics of M1 and M2 TAMs reflect the complexity of TME.

#### T lymphocytes

T cells recognize antigens on MHC molecules on the surface of antigen-presenting cells (APCs) to regulate tumor immune responses via the T cell receptor (TCR) [43]. T cells mainly include two types of CD4 + and CD8 + T cells. When MHC-II molecules are delivered to the surface of T cells, CD4 + T cells are activated and participate in the immune response by producing cytokines. CD4 + T cells with a high degree of plasticity in TME, and can be selectively different cytokine secreting cells in response to various signals [44]. Activated CD4 + T cells can be divided into various helper T cell subsets, such as Th1, Th2, Th9, Th17, Th22, Tfh and Treg [45, 46]. Each subset has its specific phenotype, cytokine profile and function. Previous studies believed that these phenotypes are mutually exclusive, and cross-phenotype expression will not occur. In fact, there have been reports of Th17Th1 cells that have both Th17 and Th1 characteristics [47]. CD8 + T cells detect antigens presented by MHC-I molecules through a cross-presentation mechanism, leading to cytotoxic reactions that lead to tumor cell death, thus called CD8 cytotoxic T lymphocytes (CTLs) [48]. After tumor infiltration, the initial CD8 + T cells differentiate into effector CD8 + T cells, and further differentiate and activate into cytotoxic and memory CD8 + T cells [49, 50]. Therefore, the two key factors for CD8 + T cells to exert anti-tumor effects are: induction of T cell differentiation Infiltrate with CD8 + T to the tumor site.

Generally speaking, in view of the cytotoxicity of CD8 + T cells, the increased number of CD8 + T cells in TME is often associated with a good prognosis [51–53]. However, CD4 + T cells have both anti- and pro-tumorigenic roles due to their different subsets and polarization [52, 54–56]. Reports in recent years have shown that in addition to antigen presentation signals and costimulatory molecules, epigenetic modification, metabolism, and iron death may also be involved in the differentiation and function of T cells [50, 57–59]. Usually, the increased number of CD8 + T cells in TME contributes to the anti-tumor response, exhaustion and dysfunction of CD8 + T cells contribute to tumor progression.

#### Blood vessels

Tumor growth and metastasis are inseparable from adequate oxygen and nutrient supply, which all depend on the tumor vasculature. Compared with normal tissues, the tumor vasculature exhibits tortuosity and dysfunction, which is reflected in the heterogeneous permeability [60]. Normally, abnormal vascular function in the tumor microenvironment leads to hypoxic environment, blocking of immune cell infiltration and low drug delivery efficiency [61–63].

Tumor vasculature mainly includes vascular sprouting, angiogenesis and vascular mimicy [64, 65]. Three important regulatory factors and their receptors are involved in the regulation of neovascularization: VEGF-VEGFR, PDGF-PDGFR and FGF-FGFR [66]. In general, VEGFR and FGFR pathways are involved in activation of endothelial cells (ECs) and maturation of blood vessels [67]. PDGFR pathway provides support for pericytes [68]. PDGFR and FGFR regulate cell migration and adhesion, and maintain the stability of blood vessel walls [66].

#### Lymphatic vessels

Tumor lymphatic vessels belong to the tumor vasculature, and their main function is to remove the interstitial fluid (ISF) formed by capillary filtrate and tissue immune surveillance [69]. Lymphatic vessels are composed of lymphatic endothelial cells (LECs) [70]. LEC signaling molecules VEGFR-3 and its ligand VEGF-C are the main driving factors of pathological lymphangiogenesis, and the extracellular protein CCBE1 and metalloproteinase Adamts3 are also involved [71–75]. Moreover, fatty acid metabolism has a regulatory effect on lymphangiogenesis [76].

The role of tumor lymphatic vessels in tumor progression has advantages and disadvantages. Certain metastatic tumors can penetrate into the lymphatic vessels and cause distant metastasis, such as breast cancer, nasopharyngeal cancer, and prostate cancer [77–79]. However, functional lymph nodes enhance the tumor's response to immune checkpoint inhibitors by reducing the ISF, which is helpful for immunotherapy [80, 81]. In addition, lymphatic vessel remodeling is closely related to tumor immunity. A recent study showed that melanoma-derived EVs deliver tumor antigens to LECs in lymph nodes for cross-presentation on MHC-I, resulting in apoptosis induction in antigen-specific CD8 + T cells [82, 83]. This also reflects the heterogeneity of lymphatic vessels in the TME.

#### Adipocytes

Adipocytes are one of the stromal cells in the TME. As a storage site for triglycerides, adipocytes not only participate in the process of energy metabolism, but also secrete various metabolites to regulate TME [84]. There are three main types of adipocytes: white adipocytes, brown adipocytes and beige adipocytes. Among them, white adipocytes are the most widely distributed adipocytes. Studies have shown that white adipose tissue (WAT) is associated with the risk of breast cancer and prostate cancer [85, 86]. In some cancer-related cachexia, the transformation of white adipocytes to brown adipocytes and atrophy may contribute to the exacerbation of cachexia [87, 88]. Brown adipocytes are cells that break down metabolic substrates such as glucose to generate heat. A new study shows that based on the dependence of cancer cells on glucose, cold exposure can activate brown fat cells to compete with cancer cells for glucose [89]. This reveals positive role of brown adipocytes in tumors. Beige adipocytes may be differentiated by a specific class of adipose progenitor cells (APCs) with plasticity [90, 91]. Beige adipocytes reduce adhesion of tumor and non-tumor mouse mammary epithelial cells, favoring tumor development [92].

Adipocytes in TME can be transformed into cancer-associated adipocytes (CAAs) after communicating with tumor cells. Compared with mature adipocytes, CAAs have smaller size, irregular shape and dispersed lipid droplets [93]. In addition to cellular morphological and structural heterogeneity, activated CAAs are associated with a reduction in markers of terminal differentiation (PPARγ, C/EBPα, HSL, APN, and FABP4) [84]. Metabolites (leptin, adiponectin, lactate, fatty acids and glutamine) secreted by CAAs can be taken up by adjacent cells in the TME to induce angiogenesis and immune escape [94, 95]. CAA-mediated secretion and processing of collagen IV induces the programming of ECM [96].

#### Stellate cells

Stellate cells originate from mesothelial and submesothelial cells with highly plasticity. The common stellate cells in the TME include hepatic stellate cells (HSCs) and pancreatic stellate cells (PSCs). Resting stellate cells are rich in vitamin A, accompanied by highly expression of desmi and glial fibrillary acidic protein (GFAP). Conversely, activated stellate cells lost vitamin A, accompanied by highly expression of α-SMA [97, 98]. Exosomes released by cancer cells can activate stellate cells, for example, exosomal miR-181a-5p and exosomal miR-21 derived from hepatic carcinoma cells can activate HSCs [99, 100]. Pancreatic cancer cells-derived exosomal miR-1290 and exosomal protein Lin28B can activate PSCs [101, 102]. Activated stellate cells can secrete VEGF, FGF, interleukins and matrix metalloproteinases (MMPs) to promote angiogenesis, inflammatory infiltration and ECM precipitation in TME [103].

##### ECM

ECM is composed of a variety of proteins and macromolecules, including collagen, glycoprotein, elastin, fibronectin and proteoglycan, which are mainly secreted by CAFs [104]. ECM has complex mechanical behaviors, and the impact of matrix stiffness on stem cells and tumor cells is a current research hotspot.

ECM has complex mechanical behavior, and the viscoelasticity and tension of the matrix can promote the stemness, metastasis and drug resistance of tumor cells [105, 106]. The increased stiffness of ECM and the remodeling of the basement membrane are conducive to tumor metastasis [107, 108]. Among them, type I collagen fibrin is very important to the stiffness of ECM. Type I collagen fibrin is a component of ECM, and its tensile strength is regulated by two enzymes: lysyl oxidases (LOXs) and lysyl hydroxylases (LHs) [109, 110]. Beyond that, the metabolism of hyaluronic acid and glucose can also lead to the remodeling of ECM components, which in turn affects cell migration [111, 112].

#### Hypoxia

Hypoxia is a hallmark of TME. Hypoxia or inadequate oxygenation is a key factor in the difficulty of eradicating tumors and also predisposes tumor cells to treatment resistance [113]. Causes of hypoxia in TME include the heterogeneity of the tumor vascular system and the exuberant metabolism of the tumor cells, where the homeostasis of oxygen supply and consumption is disrupted [114]. In hypoxia, tumor cells can activate a range of adaptive changes through hypoxia-inducible factors (HIFs). For example, hypoxia promotes the release of exosomes in a HIF-1α-dependent manner, where the exosomal miR-310a-p inhibits the ubiquitinated degradation of HIF-1α by targeting PHD3 [115]. This positive feedback loop promotes GC cell proliferation, invasion and EMT [115]. HIF-2α can induce stemness in breast cancer cells via the SOD2-mtROS-PDI/GRP78-UPRER pathway in hypoxia [116]. HIFs can enhance angiogenesis by regulating the expression of VEGF and MMP [117]. Hypoxia is associated with LOXs and LHs, the regulatory enzymes of collagen fibronectin, suggesting that hypoxia may induce changes in the physical characteristics of the ECM [118, 119]. This suggests that hypoxia is both a feature of TME and a regulatory factor.

Hypoxia can regulate exosome release. Tumor cells are more likely to release exosomes in the hypoxic TME, which is related to the activation of the small GTPase Rab27a, a major regulator of exosomal synthesis, regulated by HIF-1α [120]. It has been shown that hypoxia-treated natural killer cells secrete more exosomes compared to normoxic conditions [121]. Hypoxia may promote exosome release by regulating exosome biogenesis, which includes intraluminal vescicle (ILV) biogenesis and multivesicular endosome (MVE) transport [122, 123]. In addition to Rab27a, HIF-1α may also be involved in hypoxia-mediated exosome release through activation of Rab5a, Rab7, Rab22, RhoA, and ROCK [124–126]. Glycolysis may also be involved in the facilitation of exosomes release by hypoxia [127].

### Origin and hallmarks of exosomes

Exosomes are small extracellular vesicles (sEVs) with a diameter between 40-160 nm [12]. It originates from the endosome and is widely present in blood, urine, ascites, and cerebrospinal fluid[35]. Exosomes contain many components of cells, such as DNA, RNA, proteins, lipids and cellular metabolites [12]. Even a new study found that mitochondria from fat cells can be transported between organs through small extracellular vesicles [128]. Exosomes are internalized by receptor cells through receptor-mediated endocytosis, pinocytosis, phagocytosis, or fusion with cell membranes, resulting in the direct release of their cargo into the cytoplasm [129]. This cell-to-cell communication has been shown to change the function of recipient cells and is widespread in TME [130].

### Origin of exosomes

The specific mechanism of exosomes formation is still unclear, and the endosomal sorting complex (ESCRT) required for transportation is currently recognized as a classic pathway. The exosomes biogenesis pass through the stages of early sorting endosome, late sorting endosome, ILV and multivesicular body (MVB). Finally MVB is subsequently degraded by lysosomes or fused with the plasma membrane to release its contents, including exosomes [131]. The sorting of MVB into exosomes may have a specific regulatory mechanism, but it is currently poorly understood. It is reported that Rab27A and Rab27A, members of the GTPase Rab family, play a role in special types of secretion (such as exosome secretion and mast cell secretion) [132–134]. With the deepening of research, Rab35, Rab11 and Rab7 are also involved in the process of MVB fusion with the plasma membrane and release of exosomes [135–138]. In addition, studies have shown that actin cytoskeleton regulatory protein (cortactin) can bind to the branched actin nucleation Arp2/3 complex and further control the fusion of MVB with the plasma membrane [139].

### Characteristics and purification of exosomes

Several proteins are related to the biogenesis of exosomes, such as Rab GTPase and ESCRT protein. Exosome surface proteins including transmembrane 4 superfamily (CD9, CD63, CD81), lipid raft protein (flotillin-1), and Ceramide are often used as biomarkers for exosomes [12]. Some proteins enriched in exosomes are also commonly used as biomarkers for exosomes, such as HSP70, TSG101, and ALIX [140, 141]. Researchers can identify exosomes and their biogenesis based on these biomarkers, but there is still a lack of exosomes-directed tracking systems.

Based on the potentials of exosomes in the diagnosis and treatment of tumors, there is an urgent need for high-efficiency isolation of exosomes [142]. Density gradient differential ultracentrifugation (DGUC) has always been the most classic exosome purification method. On the basis of DGUC, an improvised one-step sucrose cushion ultracentrifugation method for exosome isolation is beneficial to maintain the integrity of exosomes and remove protein contamination [143]. In addition, many exosome separation technologies based on different principles have also been applied, such as size exclusion chromatography (SEC), ultrafiltration (UF), Anion exchange chromatography (AIEX) polymer-based precipitation, and immunoaffinity capture [144, 145]. In recent years, microfluidic-based exosome isolation techniques has been developing rapidly. Compared with traditional separation technology, microfluidic device can separate exosomes in various samples with high selectivity and high yield, while reducing processing time, cost and sample consumption [145]. Another method that uses electricity and acoustic forces to manipulate biological particles and submicron particles for deterministic sorting has been applied to the purification of exosomes. The purity of the exosomes purified by this method is more than 95% and the recovery rate is 81% [146].

### Cargos in exosomes

**Fig. 2:**
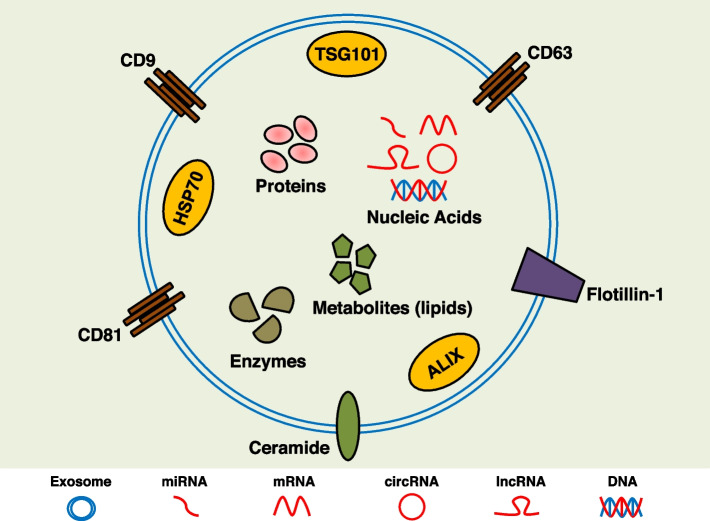
Biomarkers of exosomes and main components of exosomal cargos. Several exosome surface proteins are considered to be biomarkers of exosomes, including transmembrane 4 superfamily (CD9, CD63, CD81), lipid raft protein (flotillin-1), and Ceramide. Exosome content proteins HSP70, TSG101 and ALIX are also biomarkers of exosomes. Exosomes carry a variety of cargos, such as nucleic acids, proteins, enzymes and metabolites (mainly lipids)

Exosome cargos are the core components that confer biological effects on exosomes. Nearly 100,000 proteins and over 1,000 lipids have been reported to be associated with exosomes [147]. Nucleic acids mainly mRNAs and ncRNAs were enriched in exosomes, including more than 27,000 mRNAs and more than 10,000 ncRNAs were identified in sEV [147]. Genomic DNA (gDNA) and mitochondria DNA are present in exosomes in the form of s single-stranded or double-stranded [148, 149]. Together, these biologically active substances make up the exosomal cargos. (Fig. 2).

### Nucleic acids

The role of exosomal RNAs in tumors has been widely reported, mainly including mRNAs and ncRNAs. Regarding functional exosomal mRNAs, an early study reported that exosomal mRNAs can complete translation in receptor cells [150]. Recently, exosomal mRNAs CUL9, KMT2D, PBRM1, PREX2 and SETD2 were found to be possible novel potential biomarkers for clear cell renal cell carcinoma (ccRCC) [151]. Studies on exosomal ncRNAs have focused on miRNAs, lncRNAs & circRNAs. Usually exosomal ncRNAs are transported to the recipient cells as molecular sponges.

Exosomal DNA mainly includes gDNA and mtDNA [149]. The TME of "hibernating" cancer cells secretes EVs containing mtDNA, leading to endocrine therapy resistance in breast cancer cells [152]. T cells secrete EVs containing gDNA and mtDNA, which activate the cGAS/STING signaling pathway and induce antiviral responses in DCs [153].

### Lipids

The lipid cargos of exosomes mainly include sphingolipids, cholesterol, phosphatidylserine, saturated fatty acids, and ceramides, which are mainly associated with exosome biogenesis [154]. The bilayer lipid membrane structure of exosomes determines the enrichment of membrane lipid components (phosphoglycerolipids, sphingolipids, and sterols) [147]. The fact that neutral sphingomyelinase inhibitors can reduce exosome secretion further illustrates the importance of membrane lipids for exosomes [155]. Cholesterol enrichment in exosomes is associated with MVB. Different subcellular organelles have different cholesterol concentrations. Oxysterol-binding protein-related proteins (ORPs) are involved in cholesterol transport and are able to maintain the proper cholesterol concentration required for MVB biogenesis [156]. In addition, when low-density lipoprotein-cholesterol is low in endosomes, endoplasmic reticulum stress-derived cholesterol can be transferred to MVB [157].

Some biologically active lipids are important cargos for exosomes to perform their biological functions. LTB4 is packaged and released in exosomes [158]. This LTB-enriched exosome biogenesis originates from the nuclear envelope of centrocytes and is an unconventional pathway of exosome production [159]. Granulocyte myeloid-derived suppressor cells can secrete exosomal PGE2 to ameliorate collagen-induced arthritis [160]. Ubiquitination of 15-LO2 in hypoxia promotes 15-LO2 sorting to exosomes, which are involved in the regulation of pulmonary vascular homeostasis [161].

### Proteins

Proteins are important cargos of exosomes. Protein cargos from different sources of exosomes are heterogeneous, but the proteins involved in exosome biogenesis, exosome content sorting and exosome release are invariant. These protein cargos are as described in Sect. 2.2.2. Exosomal heterogeneity is mainly reflected in protein cargos with signal transduction function and enzyme cargos with biological activity [162]. The main exosomal signaling proteins include EGFR, VEGF, TGF-β, PTEN and STAT [163–166]. Enzymes mainly include metabolic enzymes (such as ATPase, pyruvate kinase, fatty acid synthases), RNA editing enzymes, proteases, glycosyl transferases, glycosidases [147, 167, 168]. In addition, some special exosomal proteins deserve attention. Exosomal PD-L1 is a biomarker for tumor diagnosis and immunotherapy efficacy prediction. There is glycosylation heterogeneity in exosomal PD-L1, and the sensitivity and specificity of glycosylated exosomal PD-L1 is superior compared to exosomal PD-L1 [169]. Exosomal BECN1 participated in the regulation of ferroptosis [170].

### Cargos sorting to exosomes

In general, nucleic acids can be sorted to exosomes through the interaction of RNA-binding proteins or lipid rafts in MVB [171]. Some exosomal biomarkers may regulate the process of protein sorting into exosomes [172]. The sorting of cellular metabolites may be related to the process of exosome formation [159]. In addition, there are some special mechanisms involved in the sorting of exosomal cargos. (Table 2).

**Table 2:** Different ways of various cargos sorting to exosomes

Type	Subtype	Involved molecules/mechanisms	Reference
MiRNA	miR-210	nSMase2	[173]
MiRNA	miR-17, miR-198	Sumoylated hnRNPA_2_B_1_	[174]
MiRNA	miR-486-5p	3’ end uridylated	[175]
MiRNA	miR-142-3p, miR-150, miR-451	Ago2	[176]
MiRNA	miR-181a-5p	FUS	[99]
MiRNA	miR-34c	Alyref	[177]
MiRNA	miR-155	Interaction of cRLIP and FMR1	[178]
LncRNA		lncRNA-RBP	[179]
CircRNA	circRHOBTB3	SNF8	[180]
CircRNA	CDR1as	miR-7	[181]
DNA	mtDNA	endosomal pathway	[153]
DNA	gDNA	MN	[182]
Protein	Proteins contain the KFERQ seqence	LAMP2A	[183, 184]
Protein	ECM proteins	Phosphorylated and ubiquitinated Cav1	[185]
Protein	Proteins with UBL structural domain	Act as PTM factors	[186]
Protein	HSP70-mediated proteins	Endosomal microautophagy	[187]
Metabolite	LTB4	Lyn, fotillin-1	[159]

### Nucleic acids sorting to exosomes

Initially, four mechanisms of miRNA sorting to exosomes were proposed. The neural sphingomyelinase 2 (nSMase2), heterogeneous nuclear ribonucleoprotein (hnRNP), uridylation at 3’ends and argonaute 2 (Ago2) are involved in this process [173–176]. Recent studies have shown that two RNA binding proteins, Alyref and Fus, mediate miRNA sorting into sEVs, enriching the understanding of exosomal miRNA biogenesis [99, 177]. In addition, activation of the NLRP3 inflammasome and cleavage of RILP increase exosome production, and the cleaved form of RILP interacts with FMR1 to regulate exosomal miR-155 content [178].

Some lncRNAs are also found in exosomes, probably by forming lncRNA–RBP complexes [179]. Although the specific mechanism of lncRNA sorting to exosomes is still unclear. Interestingly, lncRNA-encoded microproteins were identified in glioma-derived exosomes, indicating the biological functional diversity of exosomal lncRNAs [188].

CircRNA is a component of exosomal ncRNAs, and different circRNAs were found in exosomes originating from different cells, indicating that the sorting process of exosomal circRNAs is selective [189]. SNF8, a key component of the ESCRT-II complex, sorts circRHOBTB3 into exosomes by binding to specific sequences (141-240nt) on circRHOBTB3 [180]. After ectopic expression of miR-7 in HEK293T and MCF-7 cells, the level of circRNA CDR1as was significantly downregulated in exosomes but slightly increased in cells. This result partially suggests that sorting of circRNAs to exosomes was regulated by changes of associated miRNA levels [181].

The sorting mechanism of exosomal DNA is still unclear. Certain physiological pathways may be involved in this sorting process. MtDNA sorting to exosomes may be related to the endosomal pathway [153], and gDNA sorting to exosomes may involve micronuclei (MN) [182].

### Protein sorting to exosomes

Typically, proteins can enter cells together with cell surface proteins by endocytosis and invagination of the plasma membrane [12]. The protein sorting process can be done in organelles such as mitochondria, endoplasmic reticulum and Golgi apparatus [190]. During the budding stage of exosome biogenesis, early-sorting endosome (ESE) may fuse and communicate with mitochondria, endoplasmic reticulum and trans-Golgi network, indicating the reason why the protein can be detected in exosomes of host cells [12]. In addition, some regulatory proteins in the formation, transport and secretion of exosomes may be involved in the sorting process of exosomal proteins, such as Rab GTPase, ESCRT proteins, tetraspanins and SNARE protein complexes [172, 191–193].

Some non-canonical mechanisms are also involved in the regulation of exosomal protein sorting. A recent study shows that proteins containing the KFERQ pentapeptide sequence can be sorted to exosomes by a process dependent on the membrane protein LAMP2A, a novel mechanism independent of ESCRT [183, 184]. Epigenetic modifications can be involved in the sorting of exosomal proteins. Cav1 can be sorted to MVB and ILV in a phosphorylation and ubiquitination-dependent manner, regulates exosome biogenesis by regulating MVB cholesterol contents, and delivers specific ECM-associated proteins (Tenascin-C, fibronectin, nidogen, elilin, EDIL3 and heparan sulfate proteoglycans) to exosomes [185]. Some proteins have UBL domains (ubiquitin-like sequences), among which UBL3/MUB proteins, as one of the conserved UBLs, can act as post-translational modification (PTM) factors to regulate the process of protein sorting to sEVs [186]. In addition, endosomal microautophagy is also involved in the sorting of exosomal proteins, and the chaperone HSC70-mediated proteins are sorted into exosomes under the electrostatic interaction between the cationic domain of HSC70 and the MVB membrane [187, 194].

### Metabolites sorting to exosomes

Exosomes contain intact metabolites (mainly lipid metabolites) with characteristic of the host cells [195]. For example, exosomes secreted by granulocyte-myeloid derived suppressor cells (G-MDSCs) are enriched in PGE2, exosomes secreted by neutrophils are enriched in LTB4 [158, 160]. Triglycerides (TG) and sphingomyelin were found in T cell-derived exosomes isolated from human plasma [196]. Metabolomics of cancer stem cell-derived exosomes from melanoma revealed that multiple lipid metabolites, such as glycerophosphoglycerol (PG), glycerophosphatidylserine (PS), TG, and glycerophosphorylcholine (PC) in exosomes [197]. Currently, the mechanism by which these metabolites are sorted to exosomes remains unclear, but may be related to the interaction of lyn and fotillin-1 through the lipid domains of exosomal lipid membranes [159].

### Biology of tumor metabolic reprogramming

Metabolic abnormalities are one of the hallmarkers of tumor cells, which are metabolically reprogrammed to meet their rapid proliferation requirements [198]. Given the specific physicochemical characteristics of high pressure, high pH and hypoxia within the TME, as well as the heterogeneity of the tumor vasculature, local tumor cells often have limited metabolic resources, which accelerates the digestion of nutrients and the accumulation of metabolites. In this particular environment, tumor cells adjust their metabolism in order to maintain their growth, which not only allows them to re-meet their energy supply needs, but also regulates their gene expression and protein modifications, facilitating the spread of tumor cells [199].

### Glucose metabolism

Glucose is the main source of energy for cellular metabolism and biosynthesis. Glucose metabolism includes the glycolytic pathway, the pentose phosphate pathway (PPP), the serine synthesis pathway (SSP) and the tricarboxylic acid (TCA) cycle pathway [200]. Tumor cells are able to re-edit these pathways to obtain ATP with various biological macromolecules. In contrast to normal cells, tumor cells produce large amounts of lactate via the aerobic glycolytic pathway, resulting in an acidic TME that contributes to the proliferation [201]. In addition, increased uptake of glucose leads to the accumulation of intermediate metabolites in the glycolytic pathway, activating the cellular PPP while inhibiting the intracellular TCA[202]. Activation of the PPP provids NADPH to tumor cells, while inhibition of the TCA lead to a decrease in intracellular ROS and promoted tumor cell proliferation [203].

Heterogeneity of TME is associated with glucose metabolism. It was shown that hypoxic conditions in TME promoted HIF-1-induced glycolysis [204]. HIF-1 promotes glycolysis by upregulating hexokinase and glucose transporters, while inhibiting mitochondrial biosynthesis [204]. HIF-1 stimulates pyruvate kinase 2 (PKM2) into the nucleus to drive transcription of glycolysis-related genes [205]. HIF-1 not only regulates pH through nanohydrogen pump, but also regulates glycolysis by up-regulating hexokinase, aldolase, pyruvate kinase and downregulating pyruvate dehydrogenase to promote the conversion of pyruvate to acetyl coenzyme A, which enters the citric acid cycle [206, 207]. Abnormalities in glucose metabolism can directly affect tumor cells and non-tumor cells in TME. Up-regulated PPP in most tumor cells correlates with resistance to radiotherapy [208]. Up-regulated TCA in breast cancer cells promotes α-ketoglutarate production and facilitates tumor metastasis [209, 210]. In addition, up-regulated glycolysis in GC and NSCLC cells can regulate PD-1 expression in Treg [211]. Increased glycolysis in Treg may affect the therapeutic effect of CTLA-4 blockade, which is associated with immune infiltration in the TME [212].

### Fatty acid metabolism

Abnormal fatty acid (FA) metabolism is particularly noteworthy, as it is structural components of the membrane matrix and important secondary messengers that can serve as a fuel source for energy production [213]. In mammalian cells, FA can either be taken up directly from the surrounding environment or synthesized from scratch through nutrients. The uptake of exogenous FA requires specialized transporter proteins to achieve transmembrane, including CD36, fatty acid transporter protein (FATP) family and plasma membrane fatty acid binding protein (FABPpm), all of which are highly expressed in tumors [214]. Fat from scratch synthesis is a process that uses carbon from glucose and amino acids to convert to FA. Normally, fat from scratch synthesis is restricted to hepatocytes and adipocytes, however, tumor cells can reactivate this metabolic pathway [198].

Glucose or glutamine in tumor cells is oxidized or reversibly carboxylated by pyruvate in the TCA cycle to generate citrate, respectively [215]. Citrate is converted to acetyl coenzyme A by ATP-citrate lyase (ACLY), followed by irreversible carboxylation to malonyl coenzyme A. Finally, condensation of seven malonyl coenzyme A with one acetyl coenzyme A is catalyzed by fatty acid synthase (FASN) to produce saturated palmitic acid [213]. Palmitic acid can be converted to other FA species (like phospholipids and triglycerides) through different pathways, contributing to transmembrane signaling in tumor cells, while regulating the structure and fluidity of cell membranes and promoting epithelial-mesenchymal transition (EMT) [216].

FA contributes to the remodeling of TME. Arachidonate is an important class of bioactive lipid molecules, such as prostaglandins, leukotrienes and ω-hydroxylase [213, 217, 218]. The main enzymes involved in prostaglandin production are prostaglandin G/H synthases COX1 and COX2, are closely related to inflammation [219–221]. In addition, accumulation of FA in TME can lead to CD8 + T cell dysfunction in the pancreas [222, 223]. FA oxidation can promote IL-1β secretion by M2-type mononuclear macrophages and remodel tumor metabolism with TME [224].

### Amino acid metabolism

Amino acids are one of the raw materials for cellular synthesis of biomolecules, such as proteins, lipids, and nucleic acids. In contrast to normal cells, tumor cells require large uptake of amino acids for malignant development, as well as other amino acids to provide a source of carbon and nitrogen [225]. Reprogramming of amino acid metabolism plays an important role in tumor.

Glutamine (Gln) is one of the most abundant non-essential amino acids in the body, a recent study has shown that it is Gln that is the highest nutrient intake by cancer cells [226]. Glycolysis is the main way for tumor cells to obtain energy, and the metabolism of glucose is regulated by Gln, which is able to replace glucose as the main energy source of TCA under hypoxic microenvironment [226]. The synergistic effect between Gln and leucine can promote the breakdown of Gln to produce α-ketoglutarate and activate mTORC1,which promotes the proliferation of tumor cells [227]. Serine (Ser) is an important one-carbon unit raw material. It also serves as a methyl donor and is involved in the methylation modification of biological macromolecules [228]. Under hypoxic microenvironment, the expression of serine hydroxymethyltransferase 2 (SHMT2) was upregulated to promote the production of NADPH and glutathione to maintain redox homeostasis [229]. Tryptophan (Trp) is one of the essential amino acids and can be metabolized through three pathways: kynurenine (Kyn), 5-hydroxytryptamine (5-HT) and indole [230]. Trp may be a tumor biomarker. Trp metabolites may be biomarkers of esophageal cancer susceptibility, metastasis and prognosis [231]. Trp metabolite Kyn is overexpressed in advanced colorectal cancer and induces CD8 + T cell exhaustion [232]. Phenylalanine-tryptophan may be a combination biomarker for early diagnosis of hepatocellular carcinoma [233].

Disturbance in amino acid metabolism can remodel the immune microenvironment of tumors. It has been shown that Glutamine metabolism can regulate the immunosuppressive function of myeloid-derived suppressor cells (MDSCs) [234]. Glutamine small-molecule inhibitor not only inhibits tumor growth, but also suppresses MDSCs production and recruitment. Targeting tumor glutamine metabolism leads to a decrease in CSF3, resulting in an increase in inflammatory TAM [235, 236]. In a study of renal cancer, CAFs were found to up-regulate tryptophan 2, 3-dioxygenase (TDO) expression, resulting in enhanced secretion of Kyn, which ultimately activate AKT and STAT3 signaling pathways and induce chemoresistance [237]. (Table 3).

**Table 3:** Effects of metabolic changing on the TME

Cell	Subtype	Metabolic pathway/Melocules	Influence	Reference
Tumor cell	BC	PPP**↑**	Radioresistance**↑**	[208]
Tumor cell	BC	TCA**↑**	Metastasis**↑**	[209]
Tumor cell	GC, NSCLC	Glycolysis**↑**	Treg PD-1 expression**↑**	[211]
T cell	Treg	Glycolysis**↑**	CTLA-4 blockade therapeutic effect**↓**	[212]
Tumor cell		COX1, COX2**↑**	Inflammation**↑**	[219–221]
T cell	CD8 + T cell	FA**↑**	CD8 + memory T cell**↓**	[222]
Macrophage	M2 mononuclear macrophage	FA**↑**	IL-1β secretion**↑**	[224]
Tumor cell	CRC	Kyn**↑**	CD8 + T cell exhaustion**↑**	[232]
MDSC		Gultaminolysis**↑**	Immunosuppressive function**↑**	[234]
CAF		TDO, Kyn**↑**	Chemoresistance**↑**	[237]
CAF		Glycolysis, glutamine decomposition**↑**	Fuel adjacent cancer cells	[238]
CAF		Glycolysis, lactate**↑**	Mitophagy**↑**	[239]
CAF		GAA**↑**	ECM secretion**↑**	[240]
CAF		Acetyl-CoA**↑** citrate**↓**	CRLM**↑**	[241]
EC		ATP**↑** ROS**↓**	Maintenance of EC stability	[242]
EC	HUVEC	Glycolysis**↑**	Chemoresistance**↑**	[243]
EC		CD39, CD73, ADO**↑**	Angiogenesis, growth**↑**	[244]
T cell	Th17 cell		Th17 polarization**↑**	[245]
T cell	CD8 + T cell	IDO**↑**	CD8 + T cell exhaustion**↑**	[246]
T cell	Treg	Extracellular ATP to inosine	Inhibition of Treg function	[247]
DC		AMP hydrolysis to adenosine	Inhibition of DC function	[248]
Macrophage	M2 macrophage	OXPHOS**↑**	M2 polarization**↑**	[249]
Macrophage	M2 macrophage	CD39, CD73, ADO**↑**	M2 polarization**↑**	[244]
Macrophage	M2 macrophage	PKM2**↑**	M2 polarization**↑**	[250]
Adipocyte		TG**↓**		[251]
Adipocyte	Beige/brown adipocyte		Beige/brown differentiation**↑**	[252, 253]
Adipocyte	Beige/brown adipocyte	Lipid accumulation and glucose uptake**↑**	Beige/brown differentiation**↑**	[254]
Adipocyte		Lipolysis**↑**	Free fatty acid content**↑**	[255]
Stellate cell	PSC	OXPHOS**↑**	Mitochondrial turnover	[256]
Human adult dermal fibroblasts		Glycolysis**↑** OXPHOS**↓**	ECM acidification**↑**	[257]
Tumor cell	GC	Lipid-ROS**↓**	Ferroptosis **↓**	[258]
Tumor cell	BC	Glycolysis**↑** OXPHOS**↓**	Proliferation**↑**	[259]
Tumor cell	HCC	Glycolysis**↑**	Migration, invasion**↑**	[260]
Tumor cell	LUAD	Glutamine uptake**↑**	Tumorigenesis**↑**	[261]
Tumor cell	BC	Glycolysis**↑**	HTR**↑**	[262]
Tumor cell	BC	Glycolysis**↑** OXPHOS**↓**	Tumorigenesis**↑**	[263]
Tumor cell	Laryngeal cancer	Glycolysis**↑**	Proliferation**↑**	[264]
Tumor cell	BC	Glycolysis**↑**	Apoptosis**↓**	[265]
Tumor cell	Melanoma	FAO**↑**	Migration**↑**	[266]
Tumor cell	HCC	GLUT-1**↑**	5-FU resistance**↑**	[267]
Tumor cell	NPC	Lipid accumulation**↑**	Proliferation, migration**↑**	[268]

### Exosomal cargos-mediated metabolic reprogramming in TME

Tumor occurrence not only requires the metabolic reprogramming of cancer cells, but also the metabolic reprogramming of non-cancer cells in TME also participates in tumor progression. Exosomal cargos, as a type of intercellular communication messenger, mediate the metabolic regulation of different types of cells in TME [269, 270]. After the exosomal cargos secreted by cancer cells are accepted by recipient cells, the recipient cells undergo changes in various metabolic pathways, in which energy metabolism is reprogrammed to meet energy supply and biosynthesis. The secretion of various metabolites emphasizes the heterogeneity of TME [26, 271]. (Table 2).

### Exosomal cargos induce metabolic reprogramming of CAFs

**Fig. 3:**
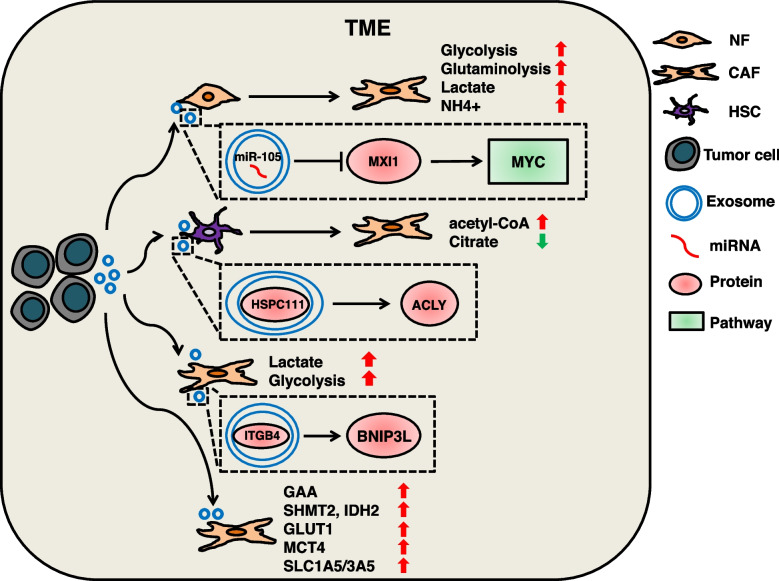
The mechanism of tumor-serected exosomal cargos regulate metabolic reprogramming of CAFs. TEXs can be delivered to NFs and stellate cells. Breast cancer cells-secreted exosomal miR-105 can target MXI1 to activate the MYC pathway and enhance the glycolysis, glutamine breakdown, lactate secretion and NH4 + clearance of CAFs. HCC cells-secreted exosomal HSPC111 can promote ACLY expression to alter lipid metabolism of CAFs. TNBC cells-secreted exosomal ITGB4 can induce BNIP3L-dependent lactate production in CAFs. CRC-derived exosomes can promote GAA of CAFs and up-regulate SHMT2, IDH2, GLUT-1, MCT4 and SLC1A5/3A5 expression

CAFs are the most common cell type in TME and the major cells producing ECM. The metabolic reprogramming of CAFs is beneficial to the growth and metastasis of tumor cells. Studies have reported that exosomal miRNAs are involved in the regulation of glucose and lipid metabolism of CAFs [100, 238]. The exosomal miR-105 secreted by breast cancer (BC) targets MXI1, activates the MYC pathway in CAFs, enhances the glycolysis and glutamine decomposition of CAFs, and detoxifies the metabolites (lactate and NH4 +) to fuel adjacent cancer cells [238]. ITGB4 is highly expressed in various cancers and contributes to tumor progression [272]. Studies on triple-negative breast cancer (TNBC) found that cancer cell-derived exosomal ITGB4 could be delivered to CAFs to induce BNIP3L-dependent mitophagy and lactate production in CAFs. Inhibition of exosomal ITGB4 delivery can inhibit mitophagy and glycolysis in CAFs [239]. Colorectal cancer (CRC) cells-derived exosomes can regulate metabolic reprogramming of CAFs, upregulation of glycogen metabolism (GAA), amino acid biosynthesis (SHMT2, IDH2) and membrane transporters of glucose (GLUT-1), lactate (MCT4), and amino acids (SLC1A5/3A5) [240]. HCC-derived exosomal HSPC111 induces differentiation of HSCs into CAFs, and exosomal HSPC111 reshapes lipid metabolism of CAFs by regulating ACLY, up-regulating acetyl-CoA levels and down-regulating citrate levels [241]. These exosome-mediated metabolic reprogramming can induce the secretory function of CAFs and enhance the heterogeneity of the TME [273]. (Fig. 3).

### Exosomal cargos induce angiogenesis through metabolic reprogramming

**Fig. 4:**
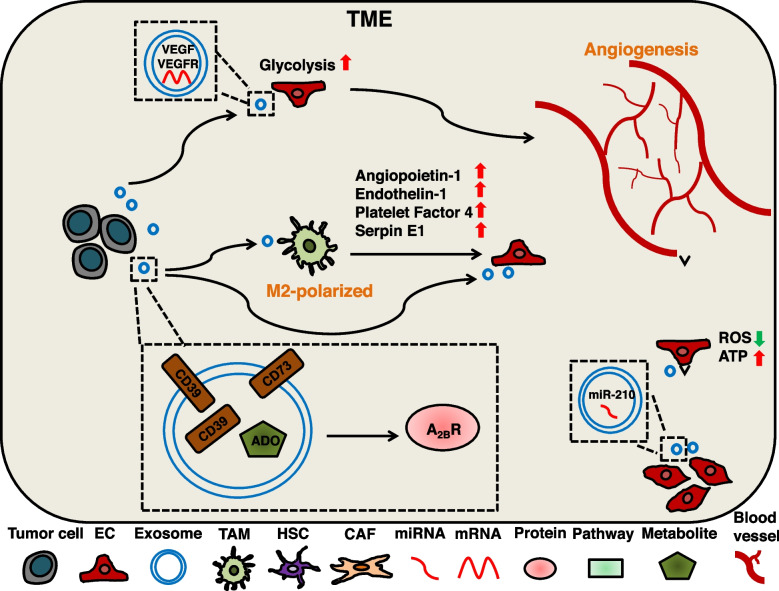
The mechanism of tumor-serected exosomal cargos induce angiogenesis through metabolic reprogramming. TEXs can be delivered to various types of cells to induce angiogenesis. AML cells-derived exosomal VEGF and VEGFR mRNA can enhance ECs glycolysis and induce angiogenesis. HNSCC cells-derived exosomal CD39, CD73 and metabolite ADO can induce A_2B_R-mediated M2-polarized TAMs and promote their secretion of angiogenic factors to induce angiogenesis. Exosomal CD39, CD73 and ADO can also directly promote ECs growth. EPC-derived exosomal miR-210 maintains vascular stability by adjusting ROS and ATP levels

Exosomal cargos are involved in the abnormal metabolism of ECs to promote tumor angiogenesis. A study has shown that endothelial progenitor cell-derived (EPC-derived) exosomal miR-210 can reduce ROS production and promote ATP production in ECs, contributing to the maintenance of ECs stability [242]. Acute myeloid leukemia (AML) cells-derived exosomes containing VEGF and VEGFR messenger RNA induce VEGFR expression in human umbilical vein endothelial cells (HUVECs). This modulation enhances glycolysis, leading to vascular remodeling and chemoresistance [243]. A study about head and neck squamous cell carcinoma (HNSCC) found that tumor cell-derived exosomes (TEXs) carry enzymatically active CD39, CD73 and adenosine (ADO), which directly induce ECs growth [244, 274]. In addition, these exosomal cargos can also induce macrophages to an angiogenic phenotype (mainly M2 polarization), the M2-polarized macrophages further secrete several pro-angiogenic factors (Angiopoietin-1, Endothelin-1, Platelet Factor 4 and Serpin E1) to stimulate the function of ECs. This dual effect (direct and indirect) promotes angiogenesis. (Fig. 4).

### Exosomal cargos induce metabolic reprogramming of immune cells

TEX-mediated metabolic reprogramming is associated with tumor immunity. It was found that purine metabolites (adenosine and inosine) enriched in TEXs may promote immune escape, and the levels of purine metabolites in circulating exosomes may suggest clinical stage and lymph node metastasis in HNSCC patients [275–277]. T lymphocytes play a key role in the tumor immune response. MiR-451 is a cell metabolism-related miRNA. Exosomal miR-451 can redistribute from gastric cancer cells with low glucose concentration to T cells and promote Th17 polarized differentiation of T cells by decreasing AMPK activity and increasing mTOR activity [245]. The cervical squamous carcinoma-derived exosomal miR-142-5p targets ARID2 to inhibit DNMT1 recruitment to the INF-γ promoter, leading to upregulation of IDO expression, thereby suppressing and exhausting CD8 + T cells [246]. TEXs can deliver a sustained signal to Treg, resulting in the conversion of extracellular ATP to inosine and inhibition of Treg function. This regulatory mechanism is dependent on surface signaling and does not require internalization of TEXs by the recipient cells [247].

**Fig. 5:**
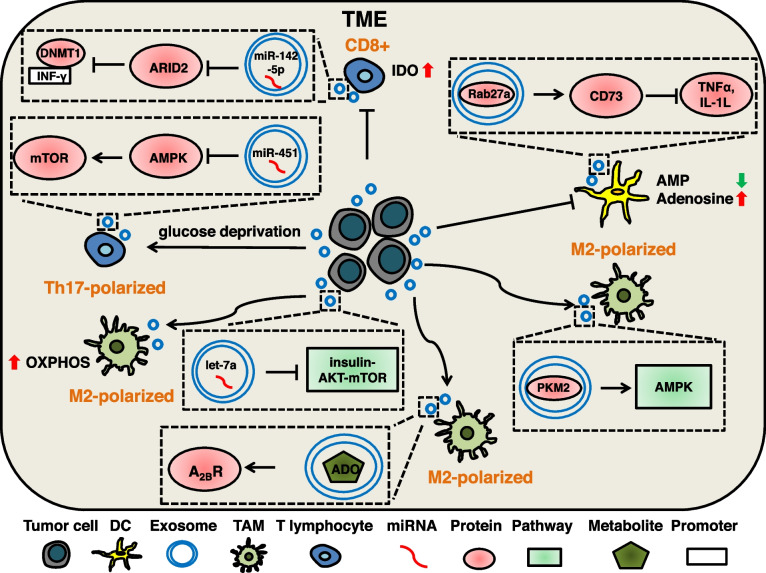
The mechanism of tumor-serected exosomal cargos regulate metabolic reprogramming of immune cells. Tumor cells-secreted exosomal miR-451 can target AMPK to activate the mTOR pathway and promote Th17 polarized differentiation of T cells through glucose deprivation. Cervical squamous carcinoma-derived exosomal miR-142-5p can target ARID2 to inhibit DNMT1 recruitment to the INF-γ promoter, leading to up-regulation of IDO, thereby suppressing and exhausting CD8 + T cells. Prostate cancer-derived exosomal Rab27a can promote CD73 expression in DCs, which hydrolyzes AMP to adenosine and inhibits the production of TNFα and IL-1L in an ATP-dependent manner, resulting in functional inhibition of DCs. Hypoxia-induced exosomal let-7a can inhibit the insulin-AKT-mTOR pathway and induce M2-polarized TAMs by enhancing OXPHOS. Lung cancer cell-secreted exosomal PKM2 can activate the AMPK pathway to induce M2-polarized TAMs, in which exosome-mediated glycolytic remodeling may play a role. HNSCC cells-derived exosomal metabolite adenosine can induce A2BR-mediated M2-polarized TAMs

Tumor antigen presentation by dendritic cells (DCs) is the initiating step of the immune response in vivo [278]. A study on prostate cancer exosomes and tumor antigen presentation found that exosomal Rab27a could induce the expression of CD73 on DCs. CD73 hydrolyzes AMP to adenosine and inhibits the production of TNFα and IL-1L in an ATP-dependent manner, which inhibits the function of DCs[248]. Exosome-mediated metabolic reprogramming induces M2 polarization in macrophages and contributes to tumor progression. Hypoxia-induced TEX can carry let-7a, and exosomal let-7a enhances OXPHOS in macrophages and inhibits the insulin-AKT-mTOR signaling pathway. This leads to M2 polarization of macrophages [249, 279]. TEX carry enzymatically active CD39, CD73 and ADO induce M2 polarization of macrophages through A_2B_R-dependent signal transduction [244]. Hypoxia is one of the features of TME, and glucose metabolism is associated with hypoxia [210]. PKM2 is one of the key enzymes of glycolysis, and studies have shown that under hypoxic conditions, lung cancer cells-derived exosomal PKM2 induces M2-polarized macrophages by activating the AMPK signaling pathway, in which exosome-mediated remodeling of glucose metabolism may play an important role [250]. Considering that the infiltration of immune cells is related to the therapeutic effect of tumors, exosome-mediated metabolic reprogramming may be an entry point for improving immunotherapy. (Fig. 5).

### Exosomal cargos induce metabolic reprogramming of adipocytes

Adipocytes in the TME can participate in tumor progression through their secretory functions, and exosomal cargos-mediated metabolic reprogramming of adipocytes can induce adipocytes to assume the CAAs phenotype. It has been shown that pancreatic cancer-derived exosomes can modulate lipid metabolism in adipocytes, in which TG is significantly down-regulated. This may be related to the increased expression of IL-6 and the promotion of lipolysis [251]. Breast cancer secreted exosomal miRNAs induce adipocyte differentiation by regulating metabolism. Exosomal miR-155 targets PPARγ and increases catabolism characterized by the release of metabolites, promoting beige/brown differentiation of adipocytes [252, 253]. Exosomal miR-126 inhibits lipid droplet accumulation and glucose uptake in adipocytes by disrupting IRS/Glut-4 signaling, and exosomal miR-144 promotes beige/brown differentiation by down-regulating the MAP3K8/ERK1/2/PPARγ axis [254]. In addition, lipolysis-inducing factors may be present in exosomes and alter the metabolism of adipocytes. Adrenomedullin (AM), which is abundant in pancreatic cancer exosomes, can promote intracellular lipolysis through the p38/ERK1/2 signaling axis and promote increased free fatty acid content in conditioned media [255].

### Exosomal cargos induce metabolic reprogramming of stellate cells

**Fig. 6:**
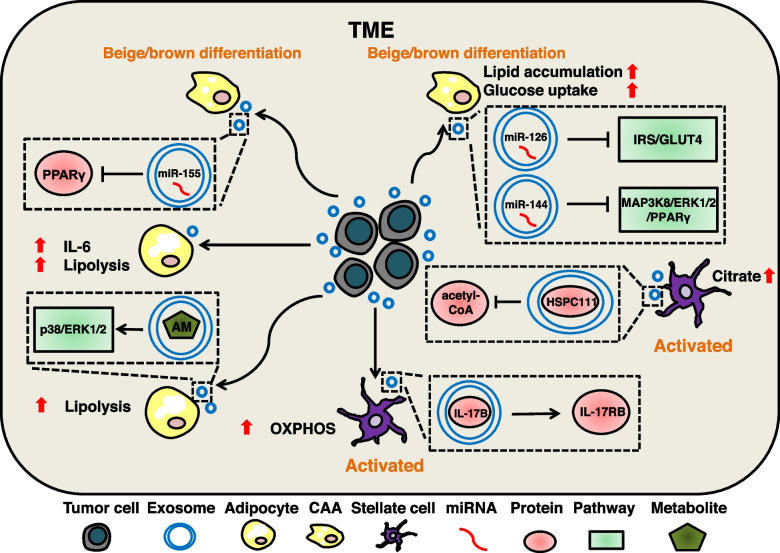
The mechanism of tumor-serected exosomal cargos regulate metabolic reprogramming of stromal cells. In addition to CAFs, adipocytes and stellate cells are the stromal cell components in TME. Pancreatic cancer-derived exosomes can reduce TG production by promoting IL-6 expression and lipolysis. AM in pancreatic cancer exosomes can activate the p38/ERK1/2 signaling axis, promote intracellular lipolysis, and increase extracellular free fatty acids. Breast cancer-secreted exosomal miR-155 can target PPARγ to promote CAAs beige/brown differentiation. Breast cancer-secreted exosomal miR-126 can inhibit the IRS/Glut-4 axis to reduce CAAs lipid droplet accumulation and glucose uptake, and exosomal miR-144 can inhibit the MAP3K8/ERK1/2/PPARγ axis to promote CAAs beige/brown differentiation. Pancreatic cancer-secreted exosomal IL-17B can activate PSCs by promotingIL-17RB expression and enhancing OXPHOS. CRC-secreted exosomal HSPC111 can activate HSCs by regulating acetyl-CoA expression, ACLY phosphorylation, and increase citrate content

Activation of stellate cells is commonly seen in liver metastasis of colorectal cancer (CRLM) and pancreatic ductal adenocarcinoma (PDAC). As part of the stroma in TME, activated stellate cells tend to have the characteristics of CAFs that facilitate the composition of the pre-metastatic niche [280]. A number of studies have shown that exosomal cargos can induce the activation of HSCs and PSCs, presenting a profibrogenic phenotype [99, 101]. However, the mechanisms of TME stellate cell activation remains unclear, and exosomal cargos-mediated metabolic reprogramming may explain this phenomenon. IL-17B secreted by pancreatic cancer can be delivered to stromal PSCs by EVs and induce the expression of IL-17RB. Up-regulation of IL-17RB in PSCs enhanced OXPHOS while reducing mitochondrial turnover to activate PSCs [256]. CRC-derived exosomal HSPC111 activates HSCs by altering the acetyl-CoA levels, citrate content and phosphorylation of ATP-citrate lyase (ACLY), causing them to exhibit a profibrogenic phenotype similar to CAFs [241]. (Fig. 6).

### Exosomal cargos remodel ECM through metabolic reprogramming

It is well known that CAFs and CAAs are the main cells that produce ECM. Exosome-mediated metabolic reprogramming can activate CAFs and CAAs in TME, which inevitably leads to an impact on ECM. Colorectal cancer (CRC) cell-derived exosomes enhance the secretion of ECM (COL1A1, Tenascin-C/X) by CAFs based on the regulation of metabolic reprogramming [240]. In addition to increased secretion, imbalance in physicochemical properties is an important manifestation of ECM heterogeneity. A study has found that human melanoma-derived exosomes are rich in exosomal miR-155 and miR-210. These exosomal miRNAs reprogram human adult dermal fibroblasts by promoting glycolysis and inhibiting oxidative phosphorylation (OXPHOS), leading to the acidification of ECM [257, 281]. The local acidification of ECM is conducive to the formation of tumor pre-metastasis niches.

### TME-derived exosomal cargos-mediated metabolic reprogramming

Exosomal cargos-mediated metabolic reprogramming is bidirectional between tumor cells and TME. Tex induces non-tumor cells in TME to exhibit a malignant phenotype, and these cells can secrete exosomal cargos to regulate metabolic reprogramming of tumor cells and accelerate tumor progression. In this way, a malignant positive feedback regulation pattern is formed between TME and tumor cells.

### CAFs-derived exosomal cargos induce metabolic reprogramming

**Fig. 7:**
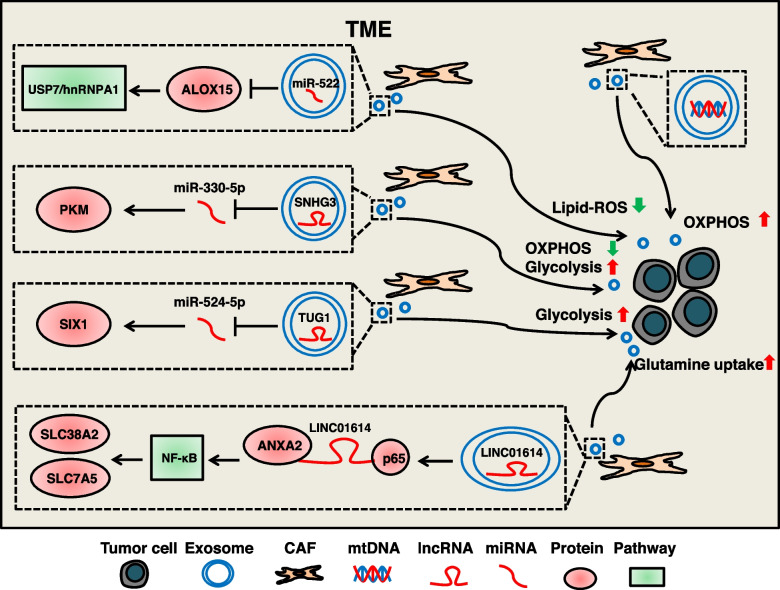
The mechanism of CAFs-derived exosomal cargos regulate metabolic reprogramming of tumor cells. CAFs regulate metabolic reprogramming of tumor cells mainly by exosomal nucleic acids. Exosomal miR-522 activates the USP7/hnRNPA1 pathway by targeting ALOX15, blocking the accumulation of lipid-ROS. Exosomal SNHG3 inhibits OXPHOS and promotes glycolysis through the miR-330-5p/PKM axis. Exosomal TUG1 promotes glycolysis through the miR-524-5p/SIX1 axis. Exosomal LINC01614 enhances tumor cells glutamine uptake by up-regulating glutamine transporters SLC38A2 and SLC7A5. In addition, exosomal mtDNA can lead to endocrine therapy resistance in OXPHOS-dependent breast cancer

Fibroblasts in the TME are activated by stimulatory signals into CAFs and remodel the TME through their secretory functions (such as CCL2, VEGF, and IL-6) [282]. The exosomal cargos secreted by CAFs can act on tumor cells and induce metabolic reprogramming, which is beneficial to tumor progression. These exosomal cargos are mainly composed of nucleic acids. (Fig. 7).

Exosomal miR-522 secreted by CAFs targets ALOX15 to activate the USP7/hnRNPA1 pathway, blocking lipid-ROS accumulation and inhibiting the ferroptosis of gastric cancer cells [258, 283]. Exosomal lncRNA SNHG3 secreted by CAFs serves as a molecular sponge for miR-330-5p in breast cancer cells. MiR-330-5p further targets PKM to inhibit OXPHOS and promote glycolysis, favoring breast cancer cells proliferation [259]. Exosomal lncRNA TUG1 secreted by CAFs promotes glycolysis in HCC cells via the miR-524-5p/SIX1 axis [260]. CAFs can regulate amino acid metabolism in lung adenocarcinoma (LUAD) cells via exosomes. CAFs-derived exosomal LINC01614 can interact with ANXA2, p65 and activate NF-κB pathway to promote the expression of SLC38A2 and SLC7A5, thus enhancing tumor cells glutamine uptake [261]. Furthermore, CAFs-derived exosomal mtDNA may regulate hormonal therapy-resistant (HTR) in breast cancer patients. A study identified mitochondrial genomes in exosomes isolated from plasma from HTR breast cancer patients. CAFs-derived exosomal mtDNA can lead to fulvestrant resistance in OXPHOS-dependent breast cancer cells [262].

### TAMs-derived exosomal cargos induce metabolic reprogramming

**Fig. 8:**
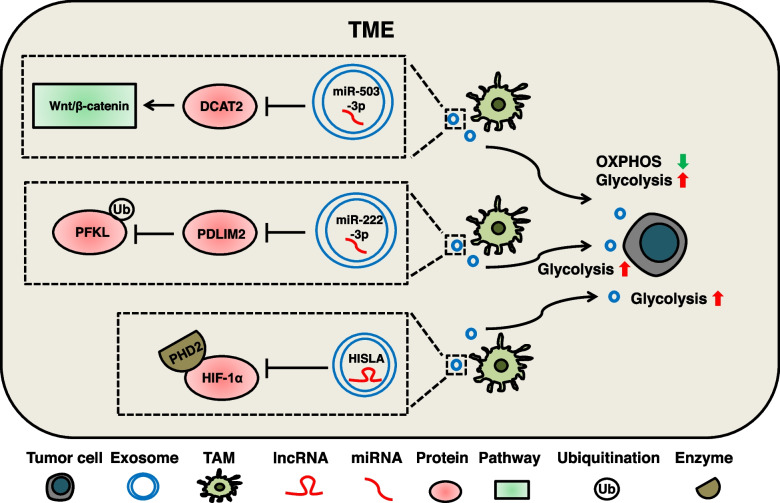
The mechanism of TAMs-derived exosomal cargos regulate metabolic reprogramming of tumor cells. TAMs-derived exosomal cargos mainly affect tumor cells glycolysis and OXPHOS. Exosomal HISLA promotes glycolysis by inhibiting the binding of HIF-1α to its hydroxylase PHD2. Exosomal miR-222-3p promotes glycolysis by targeting PDLIM2 to inhibit the ubiquitinated degradation of PFKL. Exosomal miR-503-3p activates the Wnt/β-catenin signaling pathway by targeting DACT2 to promote glycolysis and inhibit OXPHOS

Macrophages are the most numerous white blood cells in TME, and TAMs play a key regulatory role in the occurrence and development of tumors. Exosomal ncRNAs secreted by TAMs can affect the metabolic state of tumor cells [284]. In recent studies, macrophages-derived exosomal miR-503-3p targets DACT2, activates the Wnt/β-catenin signaling pathway, promotes glycolysis and reduces mitochondrial OXPHOS in BC cells [263]. M2 macrophages-derived exosomal miR-222-3p targets PDLIM2 to reduce ubiquitination of PFKL. The stabilization of PFKL promotes glycolysis in laryngeal cancer cells [264]. TAMs-derived exosomal lncRNA HISLA inhibits the degradation of HIF-1α by inhibiting the binding of HIF-1α, a key transcription factor of aerobic glycolysis with its hydroxylase PHD2. Conversely, the lactic acid secreted by tumor cells in aerobic glycolysis state can promote the sorting and loading of exosomes HISLA in macrophages [265]. This positive feedback metabolic regulation enhances the apoptosis resistance of BC cells. (Fig. 8).

### Adipocytes-derived exosomal cargos induce metabolic reprogramming

**Fig. 9:**
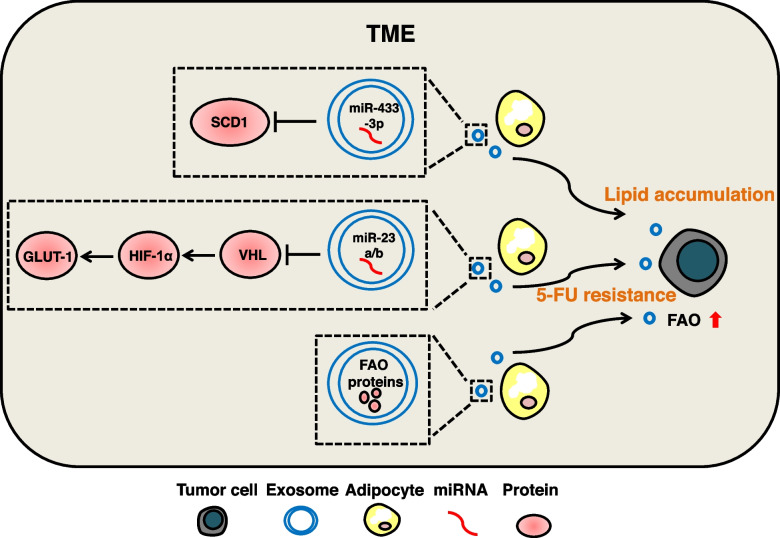
The mechanism of adipocytes-derived exosomal cargos regulate metabolic reprogramming of tumor cells. The effect of adipocytes-derived exosomal cargos on tumor cells metabolism is reflected in lipid. Some FAO-associated proteins are enriched in adipocytes-derived exosomes and promote FAO in tumor cells via exosomes. Exosomal miR-433-3p targets SCD1 to promote lipid accumulation in tumor cells. Exosomal miR-23a/b targets VHL to promote HIF-1α expression, which enhances GLUT-1 expression and 5-FU resistance in tumor cells

Previous studies on exosomes and stromal cells in TME have mostly focused on CAF, but little attention has been paid to CAA, especially the exosomes secreted by CAAs and their functions. In fact, adipocytes, as a kind of secretory cells, can not only secrete metabolites such as leptin and fatty acids directly, but also secrete bioactive cargos in the form of exosomes, which can modify the metabolism of tumor cells. Advanced melanoma cells are in direct contact with subcutaneous adipocytes. Adipocytes can directly transfer fatty acids into melanocytes, resulting in increased lipid content and abnormal lipid droplet formation. [285]. In addition to direct contact, exosomes act as a bridge between melanoma and adipocytes. Mass spectrometry showed that adipocytes-derived exosomes were rich in fatty acid oxidation (FAO) related proteins, which promoted FAO and migration of melanoma cells. [266]. This suggests that adipocytes-derived exosomes may induce metabolic reprogramming of tumor cells, the mechanism remains to be elucidated [286]. Subsequently, it was found that the adipocyte-secreted exosomal miR-23a/b regulates GLUT-1 expression by targeting the VHL/HIF-1α axis, leading to 5-FU resistance in HCC cells [267]. A recent study found that the adipocyte-derived exosomal miR-433-3p in a hypoxic environment can target SCD1 (a key regulatory gene for the synthesis of monounsaturated fatty acids) to promote lipid accumulation in NPC cells and facilitate proliferation and migration [268]. (Fig. 9).

### Exosome-based tumor metabolic therapies

As oncology researches have become more detailed, the understanding of tumors has transitioned from genetic diseases to chronic metabolic diseases. The aim of anti-tumor therapy has also gradually evolved from targeting molecular biomarkers to targeting tumor metabolic pathways. The development of single-cell sequencing technology has led to a more comprehensive understanding of TME and the discovery that metabolic reprogramming exists not only in tumor cells, but also in other components of TME (stromal cells, immune cells and endothelial cells) [287, 288]. These non-tumor cells have adapted to TME by altering metabolic pathways, creating a pre-metastatic niche that favors tumor progression [289]. It is hopeful to target specific exosomal cargos-mediated metabolic pathways and develop exosome-based vehicles for anti-tumor therapy. (Table 4).

**Table 4:** Therapeutics targeting exosome-mediated metabolism

Drug/Method	Tumor	Therapeutic target	Therapeutic effect	Reference
Shikomin	NSCLC	Exosomal PKM2**↓**	Cisplatin sensitivity**↑**	[290]
Shikomin	Bladder cancer	Exosome release**↓**	Cisplatin sensitivity**↑**	[291]
Exosome-transported si-ciRS-122	CRC	Glycolysis**↓**	Oxaliplatin sensitivity**↑**	[292]
Coptisine	HCC	Exosomal circCCT3**↓**	Proliferation, invasion**↓**	[293]
DHA	BC	Exosomal miR-101, miR-9, miR-342**↑** miR-382, miR-21**↓**	Angiogenesis**↓**	[294]
Exosome-mediated cPLA2 siRNA	GBM	Energy metabolism**↓**	Proliferation**↓**	[295]
TEX-mediated AIEgens and PPI	GC	Glutamine metabolism**↓**	PDT**↑**	[296]

The most concise way to target exosomal cargos-mediated reprogramming of TME metabolism for tumor treatment is to inhibit exosomal secretion. PKM2 is a key enzyme in glycolysis and is involved in the secretion of exosomes in addition to directly reshaping cellular metabolism through the OXPHOS and Warburg effects [297]. During the release of exosomes, phosphorylated PKM2 acts as a protein kinase to promote the formation of SNARE complexes by enhancing the phosphorylation of SNAP23 [298]. During the release of exosomes, phosphorylated PKM2 acts as a protein kinase to promote the formation of SNARE complexes by enhancing the phosphorylation of SNAP23. PKM2 combines metabolic regulation with non-metabolic regulation of exosome secretion, is an ideal target for exosome and tumor metabolic therapy. Shikonin is the active ingredient of Comfrey, a naphthoquinone compound [290]. Shikonin is a specific PKM2 inhibitor that not only inhibits glucose uptake and lactate production in tumor cells, but also inhibits glycolysis by reducing extracellular secretion of exosomal PKM2 and enhances cisplatin sensitivity in NSCLC cells [290]. A study on bladder cancer found that highly-expression of PKM2 was associated with cisplatin resistance. Shikonin can promote cisplatin sensitivity of bladder cancer cells by reducing the release of exosomes, but the specific mechanism remains to be explored [291].

Exosomal ncRNAs, as common cargos in TME, play an important role in the metabolic reprogramming of TME. It was found that oxaliplatin-resistant CRC cells-derived exosomal circRNA ciRS-122 was delivered to sensitive cells, which enhanced glycolysis and chemoresistance in sensitive cells via miR-122/PKM2 signaling axis [292]. Development of exosome-transported si-ciRS-122 can reverse the ciRS-122/miR-122/PKM2 signaling axis to inhibit glycolysis and enhance chemosensitivity in CRC cells. In addition to tumor cells, targeting exosomal circRNAs derived from CAFs in TME has antitumor effects. It was found that exosomal circCCT3 secreted by CAFs could enhance glucose metabolism by regulating the expression of HK2. It was found that exosomal circCCT3 secreted by CAFs could enhance glucose metabolism by regulating the expression of HK2. Treatment of CAFs with coptisine inhibited the secretion of exosomal circCCT3 and suppressed HCC cell proliferation and invasion [293]. In addition, docosahexaenoic acid (DHA) as an omega 3 free fatty acid has been reported to exert anti-angiogenesis effects. DHA can alter the expression of angiogenesis-related exosomal miRNAs in breast cancer cells, inhibits angiogenesis by up-regulating exosomal miR-101, miR-199, and miR-342, and down-regulating exosomal mir-382 and miR-21 to exert anti-tumor effects [294, 299].

Benefiting from the targeting and biocompatibility of exosomes, exploitation of exosomes as carriers for drug delivery targeting tumor metabolism has a bright future. Although there are currently no engineered exosomes to directly target various metabolic pathways in the TME, exosomes can be combined with classical drugs or modalities as an adjuvant therapy to improve the efficacy of anti-tumor therapy. It has been shown that combination of exosome-mediated cPLA2 siRNA and metformin reduces the growth of glioblastoma xenografts by impairing the energy metabolism of mitochondria [295]. Photodynamic therapy (PDT) is a novel method of treating tumors with photosensitizing drugs and laser activation [300]. Aggregation-induced emission luminogens (AIEgens) are photosensitizers for PDT whose efficacy is limited by GSH. A recent work developed TEX for the co-delivery of AIEgens and proton pump inhibitor (PPI) for tumor combination therapy. TEX can specifically deliver AIEgens and PPI to tumor sites, and PPI inhibits GSH and ATP produced by glutamine metabolism in tumor cells, which contribute to the efficacy of AIEgens [296]. The combination of exosomes, glutamine metabolism and PDT may be a new option for future tumor treatment, but treatments that inhibit glutamine metabolism still need to be approached with caution. Glutamine depletion may stimulate release from Rab11a compartments of exosomes with pro-tumorigenic functions [301]. Therefore, exosome-based tumor metabolic therapy still needs further refinement to find the balance between pro-tumorigenesis and anti-tumorigenesis.

## Conclusion

This review highlights the multiple roles and molecular mechanisms of exosome-mediated metabolic reprogramming in TME reprogramming. The field of exosomes (or EVs) has made great progress in recent years benefiting from technological breakthroughs in isolation, purification, in vivo tracking and content analysis [2]. This has led to the identification of other types of EVs besides exosomes and their functions becoming a novel hotspot in the field of EVs. In the future, the understanding of exosomes will be enriched by how to precisely distinguish exosomes from other EVs subtypes and exclude contaminants to further obtain high purity exosomes. This will also help to improve the targeting and biosafety of antitumor therapies developed with exosomes as vectors.

We describe the cell–cell communication mediated by exosomal cargos in TME and how these cargos are sorted to exosomes. Along with technological advances, the way of sorting various types of cargos into exosomes (or specific subtypes of EVs) is the bottleneck for further development in the field of exosomes. The bioactive cargos are the key to the function of exosomes. In addition to the cell-derived exosomal cargos in human TME, milk exosomes have a bright future as an oral drug delivery system, due to the biocompatibility of milk exosomes with exogenous cargos [302].

The heterogeneity of TME promotes tumor proliferation, metastasis, stemness and drug resistance. We summarized the main components and characteristics of TME, and highlighted the role and mechanism of exosomal cargos-mediated metabolic reprogramming in the heterogeneity of TME. Improving TME becomes an emerging strategy for anti-tumor treatment. The plasticity of tumor metabolism is both promising and challenging. Given the complex composition of TME, targeting one component for metabolic remodeling is difficult, and we need to consider more whether altered metabolism has the same therapeutic effects on multiple components of TME. Application of tumor organoid platforms to exosomes may be used to simulate the effect of exosomes on TME.

Exosomal cargos-mediated abnormalities metabolism in TME remains to be extensively studied. Considering the widespread of exosomal cargos, exploring the molecular mechanisms of exosomal cargos-induced metabolic reprogramming is beneficial for tumor precision treatment. As more and more biologic companies are entering the exosome field, the development of exosome-based drug delivery modalities to reshape metabolism in TME is promising. Combining chemotherapy, radiotherapy or targeted therapy with novel metabolic therapies may be the future trend in tumor treatment.

## Data Availability

Not applicable.

## Abbreviations

TME: Tumor microenvironment
EVs: Extracellular vesicles
MVs: Microvesicles
ncRNA: Non-coding RNA
CAFs: Cancer-associated fibroblasts
ECM: Extracellular matrix
NFs: Normal fibroblasts
HGSOC: High-grade serous ovarian cancer
TAMs: Tumor-associated macrophages
MDMs: Monocyte-derived macrophages
RCC: Renal cell carcinoma
APCs: Antigen-presenting cells
TCR: T cell receptor
CTLs: Cytotoxic T lymphocytes
ECs: Endothelial cells
ISF: Interstitial fluid
LECs: Lymphatic endothelial cells
WAT: White adipose tissue
CAAs: Cancer-associated adipocytes
HSCs: Hepatic stellate cells
PSCs: Pancreatic stellate cells
LOXs: Lysyl oxidases
LHs: Lysyl hydroxylases
HIFs: Hypoxia-inducible factors
ILV: Intraluminal vescicles
MVE: Multivesicular endosome
sEVs: Small extracellular vesicles
ESCRT: Endosomal sorting complex
MVB: Multivesicular body
MMPs: Matrix metalloproteinases
DGUC: Density gradient differential ultracentrifugatio
SEC: Size exclusion chromatography
UF: Ultrafiltration
AIEX: Anion exchange chromatography
gDNA: Genomic DNA
ccRCC: Clear cell renal cell carcinoma
OPRs: Oxysterol-binding protein-related proteins
nSMase2: Neural sphingomyelinase 2
hnRNP: Heterogeneous nuclear ribonucleoprotein
Ago2: Argonaute 2
MN: Micronuclei
ESE: Early-sorting endosome
PTM: Post-translational modification
G-MDSCs: Granulocyte-myeloid derived suppressor cells
TG: Triglycerides
PG: Glycerophosphoglycerol
PS: Glycerophosphatidylserine
PC: Glycerophosphorylcholine
PPP: Pentose phosphate pathway
SSP: Serine synthesis pathway
TCA: Tricarboxylic acid
PKM2: Pyruvate kinase 2
FA: Fatty acid
FATP: Fatty acid transporter protein
FABPpm: Plasma membrane fatty acid binding protein
ACLY: ATP-citrate lyase
FASN: Fatty acid synthase
EMT: Epithelial-mesenchymal transition
Gln: Glutamine
Ser: Serine
Trp: Tryptophan
Kyn: Kynurenine
5-HT: 5-Hydroxytryptamine
MDSCs: Myeloid-derived suppressor cells
TDO: Tryptophan 2, 3-dioxygenase
BC: Breast cancer
HCC: Hepatocellular carcinoma
TNBC: Triple-negative breast cancer
CRC: Colorectal cancer
AML: Acute myeloid leukemia
EPC: Endothelial progenitor cell
HUVECs: Human umbilical vein endothelial cells
HNSCC: Head and neck squamous cell carcinoma
TEXs: Tumor cell-derived exosomes
ADO: Adenosine
DCs: Dendritic cells
AM: Adrenomedullin
CRLM: Metastasis of colorectal cancer
PDAC: Pancreatic ductal adenocarcinoma
OXPHOS: Oxidative phosphorylation
LUAD: Lung adenocarcinoma
HTR: Hormonal therapy-resistant
FAO: Fatty acid oxidation
NSCLC: Non-small cell lung cancer
DHA: Docosahexaenoic acid
PDT: Photodynamic therapy
PPI: Proton pump inhibitor
